# The Association Between Serum 25-Hydroxyvitamin D Levels and Patients With Allergic Rhinitis

**DOI:** 10.7759/cureus.9762

**Published:** 2020-08-15

**Authors:** Afnan F Bukhari, Mohammed J. Felemban, Hesham Alem

**Affiliations:** 1 Otolaryngology - Head and Neck Surgery, King Abdulaziz University Hospital, Jeddah, SAU; 2 Otolaryngology - Head and Neck Surgery, King Abdulaziz University, Jeddah, SAU

**Keywords:** ar: allergic rhinitis, ksa:kingdom of saudi arabia, ige, immunoglobulin e, sino-nasal outcome test-20 (snot-20)

## Abstract

Introduction

Many studies have suggested a link between vitamin D deficiency and the development of other atopic diseases like allergic rhinitis (AR). AR can lead to sleep disturbance, fatigue, depressed mood, and compromised cognitive function, which can impair the quality of life and productivity in many people.

Objective

We aimed to determine the association between vitamin D levels and AR and the effect of vitamin D on atopy markers.

Methods

All patients with AR who were diagnosed, treated, and followed up at the King Abdulaziz University Hospital (KAUH), Jeddah, Saudi Arabia from January 2012 to January 2020 were included in the study. Our exclusion criteria were as follows: pediatric patients, patients with insufficient follow-up data, patients with no atopy markers, patients with comorbid conditions affecting their serum vitamin D levels, and patients with a history of taking medications that affect serum vitamin D levels.

Results

Fifty-five adult patients with AR were included in the study. Patients with vitamin D deficiency were more likely to have uncontrolled AR. Regarding the effect of vitamin D deficiency on atopy markers, there was no statistically significant relationship between vitamin D deficiency and serum immunoglobulin E (IgE) levels. However, serum eosinophil levels were significantly higher in patients with vitamin D deficiency.

Conclusion

Our results showed that vitamin D deficiency is strongly associated with uncontrolled AR; there was a statistically significant relationship between vitamin D deficiency and eosinophil levels, but no significant relationship between vitamin D deficiency and serum IgE was found.

## Introduction

Allergic rhinitis (AR) is a symptomatic disorder of the nose that is induced when the immune system overreacts to allergens in the air, leading to immunoglobulin E (IgE)-mediated inflammation of the membranes lining the nose [1]. Signs and symptoms of this condition can include rhinorrhea with thin, watery nasal discharge associated with postnasal drip, nasal congestion, sneezing, itchy nose and roof of the mouth, itchy ears and/or palate, and itchy red eyes [2].

AR can lead to sleep disturbance, fatigue, depressed mood, and compromised cognitive function, which can impair the quality of life and productivity in many people. The symptoms of AR can lead to absences from work or school [2]. Moreover, AR can predispose the patients to sinusitis and nasal polyps, and ear infection [3]. Triggers for AR could be domestic allergens, such as mites, insects, or outdoor allergens of plant origin, including pollens and molds. In addition, occupational triggers could play a part in causing AR; these include latex, tobacco smoke, automobile exhaust, ozone, and nitrogen oxide [4]. AR is a global health problem that causes considerable economic and societal burdens. Between 10-30% of the world’s population suffer from AR.

Vitamin D, which is essential for strong bones, helps the body use calcium from the diet. The deficiency of vitamin D is responsible for the development of rickets in children and osteomalacia in adults [5]. Vitamin D obtained from sun exposure and food and dietary supplements is biologically inert and must undergo activation by two hydroxylation processes in the body. The first occurs in the liver, converting vitamin D to 25-hydroxyvitamin D [25(OH)D], also known as calcidiol. The second occurs primarily in the kidney and forms the physiologically active 1,25-dihydroxyvitamin D [1,25(OH)2D], also known as calcitriol [6,7].

Vitamin D is known to be important for skeletal integrity. Some studies in adults have shown that vitamin D is associated positively with bone mineral density. In addition, studies in children and adolescents have reported that optimal vitamin D level is essential to attain peak bone mass [7]. There is no absolute consensus on the cut-off value between a normal and low level of vitamin D. Many studies have used 32 ng/ml as a cut-off value, and most experts now suggest that the normal level of 25(OH)D is ≥30 ng/ml (75 nmol/l). There has been a consensus among these experts, and based on that, a level of >20-29 ng/ml (50-75 nmol/l) is considered vitamin D insufficiency, and a level of ≤20 ng/ml is associated with vitamin D deficiency [8]. 

Saudi Arabia is one of the sunniest regions of the world, and exposure to sunlight might be assumed to be sufficient to maintain adequate vitamin D status. However, vitamin D deficiency is common among the Saudi population [9]. As early as 1982, Woodhouse and Norton reported low vitamin D levels in the ethnic Saudi Arabian population [10]. Later, Sedrani confirmed these findings and also found that low vitamin D levels were not related to any specific region, sex, age, or season [11].

Despite these reports, no action was taken to fortify foods adequately or to encourage people to increase their vitamin D intake. Studies investigating vitamin D status among adults in the Saudi Arabian population have been scarce, and to date, none of them have studied a sample that can be regarded as nationally or geographically representative. The currently available literature on the Saudi Arabian population suggests that vitamin D deficiency is around 60% and more common in females and people of a young age [12].

Recently, based on the important role of vitamin D in the immune system, many studies have reported that vitamin D may be implicated in the development of AR. However, the findings are contradictory, since some studies have found a significant association while others found a negative association [13-15]. Therefore, this study aims to explore the role of vitamin D in AR patients in Saudi Arabia.

The aim of this study is to determine if there is a direct relation between AR and the serum levels of 25(OH)D and to detect the levels of serum IgE and serum eosinophils in patients in our study group.

Allergic rhinitis in Saudi Arabia

Although the observed prevalence of AR in Middle Eastern countries is low compared to Western countries, its burden is still considerable. AR in general, and uncontrolled and severe forms of it in particular, results in a negative impact on the quality of life, quality of sleep, and daily activities [16]. AR, with or without asthma, is a disorder that troubles many adults and children. The prevalence of allergic and hypersensitive reactions has increased with the developing and rapidly changing nature of Saudi Arabia’s environment, which has been associated with imported flora, new classes of industrial and household chemicals, consumption of a variety of processed foods and drinks, and the wide use of synthetic materials [17,18].

A large body of data has been recorded in Saudi Arabia that proves the importance of pollen sensitization, which is attributed to irrigation and the increased local implantation of trees and flowers. In a preliminary study, Marglani et al. performed a skin prick test (SPT) study using 24 allergen extracts on 112 patients with AR and asthma at a tertiary care center. Eighty-two patients (73.2%) had a positive skin prick test. Dust mite Dermatophagoides pteronyssinus (DP) and Dermatophagoides farinae (DF) were found to be the most common allergens, and Mimosa was found to be the least common one. In the exclusive AR group, the most common allergen was dust mite DF [19].

AR is two to four times more common than occupational asthma and more frequently precedes the development of asthma [20]. In 1987, Al-Nahdi and Al-Quorain performed SPTs in 210 allergy patients in the city of Dammam in eastern Saudi Arabia [21]. Fifty-five patients (26.2%) had at least one positive SPT and most were polysensitive.

Vitamin D deficiency in Saudi Arabia

A meta-analysis involving 16 published studies was conducted between 2008 and 2015 about vitamin D deficiency in Saudi Arabia to find the average levels of vitamin D deficiency in Saudi Arabian population. A total of 20,787 patients were analyzed; 62% (12,959) were females, and the rest were males. The currently available literature on the Saudi Arabian population suggests that vitamin D deficiency stands at around 60% [22].

## Materials and methods

Study design

The study protocol was reviewed and approved by the Institutional Review Board committee at the King Abdulaziz University Hospital (KAUH), Jeddah, Saudi Arabia. Ethical approval for this study was obtained from the Research Ethics Committee at KAUH. A retrospective cohort study was designed to determine the association between vitamin D levels and AR and the effect of vitamin D on atopy markers.

Patients and setting

All patients with AR who were diagnosed, treated, and followed up at KAUH From January 2012 to January 2020 were included in the study. All included patients underwent comprehensive otorhinolaryngology-head and neck clinical examination, including nasal scopes and atopy markers. Data were collected from the participant using a pre-validated questionnaire administered in the outpatient clinic [Sino-Nasal Outcome Test (SNOT-20)]. The exclusion criteria were as follows: pediatric patients, patients with insufficient follow-up data, patients with no atopy markers, patients with bronchial asthma and comorbid conditions affecting their serum vitamin D levels, e.g., cystic fibrosis, osteomalacia, sarcoidosis, Crohn's disease, celiac disease, and patients with a history of taking medications that affect serum vitamin D levels, e.g., corticosteroids, bisphosphonates, and vitamin D supplements.

Processing of medical records

All information obtained during the study was kept confidential. Subjects’ personal identities were kept confidential by assigning an identification number to each participant. Subjects' names and other personal identifying information will not be used in any reports, presentations, or publications. Personal information will be kept strictly confidential, stored in a computer that is password-locked, and only accessed by the principal investigators. As the main objective of this study was to determine the relationship between AR and vitamin D deficiency, blood samples were drawn from the patients to measure their vitamin D levels, serum IgE, and serum eosinophils. Vitamin D levels were measured by enzyme-linked immunosorbent assay. Patients were separated into three categories based on their vitamin D levels: optimal vitamin D levels [>75 nmol/l (30 ng/ml)], insufficient vitamin D [50-75 nmol/l (>20-29 ng/ml)], and deficient vitamin D levels [<50 nmol/l (<20 ng/ml)]. Patients were categorized according to the Allergic Rhinitis and its Impact on Asthma (ARIA) guidelines. The severity of nasal symptoms was assessed using a visual analog scale - mild: 0-3; moderate: 4-7; severe: 8-10.

The data collected were entered and analyzed using SPSS Statistics version 23.0 (IBM, Armonk, NY) for Mac OS; statistical analyses included descriptive and inferential statistics. Descriptive statistics included frequencies, percentages, mean, median, and standard deviations; the inferential statistic test used was the chi-square test. This test was run to determine whether there was a statistically significant relationship between AR and vitamin D deficiency and if there was a statistically significant association between the patient’s age, gender, and the main variables of the study. A p-value of <0.05 was considered statistically significant.

## Results

Patient demographic characteristics

A total of 55 patients were included in the study, and the general characteristics of this study population are presented in Table 1. Of the 55 participants, 37 were females and 18 were males (32.7%); the mean age of the participants was 42 years (range: 18-80 years). All patients were further tested for total and allergen-specific serum IgE levels, serum vitamin D, and serum eosinophil levels.

**Table 1:** Demographic characteristics of the study population IgE: immunoglobulin E; SD: standard deviation

Characteristics	Frequency Percentage			
Gender				
Male	18 32.7			
Female	37 67.3			
Age groups, years	Frequency	Percentage	Mean	SD
19-39	30	54		
40-59	15	27	42.0	15.66
60-70	7	12.7		
> 70	3	5		

Classification of patients according to clinical characteristics 

Out of the 55 patients, 38.2% had moderate AR, while 32.7% had severe AR, and 29.1% had mild disease. The majority of patients (74.5%) included in this study were vitamin D-deficient, 20% had insufficient levels of vitamin D, and only 5.5% of the patients had optimal levels of vitamin D. Regarding the level of serum IgE levels in patients with AR, the study found that the majority of the patients had normal (49.1%) or high (49.1%) levels. Only one patient was detected to have low levels of IgE. The level of serum eosinophils among patients showed that 67.4% had a normal level of serum eosinophils, while 20% of the patients (n=11) had a high level of serum eosinophils. Only 3.6% of the patients (n=2) had low levels of serum eosinophils; the patients' clinical characteristics are presented in Table 2.

**Table 2:** Clinical characteristics of participants with total and allergen-specific serum IgE, vitamin D, and eosinophils measurements IgE: immunoglobulin E; SD: standard deviation

Characteristics	Frequency	Percentage	Mean	SD
Vitamin D level				
Low	41	74.5		
Normal	11	20	1.31	0.57
High	3	5.5		
IgE level				
Low	1	1.8		
Normal	27	49.1	2.47	0.54
High	27	49.1		
Eosinophil level				
Low	2	3.6		
Normal	42	76.4	2.16	0.46
High	11	20		

The impact of serum vitamin D levels among allergic rhinitis patients

To examine the relationship between vitamin D deficiency and AR, a chi-square test was applied. It was found that severe vitamin D deficiency was significantly higher in those with severe AR (p: <0.001), as shown in Table 3. This indicates that there is a statistically significant relationship between vitamin D deficiency and AR among patients.

**Table 3:** The impact of serum vitamin D levels among allergic rhinitis patients *The value of the chi-square test is equal to 18.654, which is statistically significant at p=0.001 level

		Allergic rhinitis				Chi-square	df	P-value
		Mild, n (%)	Moderate, n (%)	Severe, n (%)	Total, n (%)			
Vitamin D level	Low	16 (29.1%)	10 (18.2%)	15 (27.3%)	41 (74.5%)	18.654*	4	0.001
	Normal	0 (0.0%)	10 (18.2%)	1 (1.8%)	11 (20.0%)			
	High	0 (0.0%)	1 (1.8%)	2 (3.4%)	3 (5.5%)			
	Total	16 (29.1%)	21 (38.2%)	18 (32.7%)	55 (100.0%)			

Differences in mean serum 25(OH)D levels based on the presence of elevated serum IgE levels

We wanted to determine whether serum 25(OH)D levels correlated with IgE levels by comparing the estimated mean values. Without adjusting for potential confounders, although the mean 25(OH)D levels were lower in participants with normal and increased serum IgE levels, the results showed no statistically significant relationship between mean serum vitamin D levels and serum IgE as the contingency coefficient value was not significant (p: >0.05) (Table 4).

**Table 4:** Differences in mean serum 25(OH)D levels based on the presence of elevated serum IgE levels 25(OH)D: 25-hydroxyvitamin D; IgE: immunoglobulin E

		Serum IgE				Contingency coefficient	P-value
		Low, n (%)	Normal, n (%)	High, n (%)	Total, n (%)		
Vitamin D level	Low	1 (1.8%)	20 (36.4%)	20 (36.4%)	41 (74.5%)	0.267	0.38
	Normal	0 (0.0%)	7 (12.7%)	4 (7.3%)	11 (20.0%)		
	High	0 (0.0%)	0 (0.0%)	3 (5.5%)	3 (5.5%)		
	Total	1 (1.8%)	27 (49.1%)	27 (49.1%)	55 (100.0%)		

Differences in mean serum 25(OH)D levels based on the presence of elevated serum eosinophil levels

Analyses of serum eosinophil levels among patients showed that 67.4% had a normal level of serum eosinophils, while 20% of the patients (n=11) had a high level of serum eosinophils. Only 3.6% of the patients (n=2) had a low level of serum eosinophils. Serum eosinophil levels were significantly higher in patients with vitamin D deficiency (p=0.04), as shown in Table 5.

**Table 5:** Differences in mean serum 25(OH)D levels based on the presence of elevated serum eosinophil levels *Indicates that the chi-square test is statistically significant at p=0.04 level 25(OH)D: 25-hydroxyvitamin D

		Serum eosinophil				Chi-square	df	P-value
		Low, n (%)	Normal, n (%)	High, n (%)	Total, n (%)			
Vitamin D level	Low	1 (1.8%)	30 (54.5%)	10 (18.2%)	41 (74.5%)	9.884*	4	0.04
	Normal	0 (0.0%)	10 (18.2%)	1 (1.8%)	11 (20.0%)			
	High	1 (1.8%)	2 (3.6%)	0 (0.0%)	3 (5.5%)			
	Total	2 (3.6%)	42 (76.4%)	11 (20.0%)	55 (100.0%)			

This result indicates that there is a statistically significant relationship between vitamin D deficiency and elevated serum eosinophil levels.

Differences in mean serum 25(OH)D levels based on the patients' demographic data including age and gender

To examine if there was a statistically significant association between vitamin D deficiency and patients’ gender and age, a chi-square test was performed. There was no statistically significant association as the contingency coefficient value was not significant (p: >0.05).

## Discussion

Visual analog scales have been used for measuring the severity of symptoms in several diseases in recent years. They have been tested and validated for AR as well; this approach, as advised by the new ARIA guidelines, is useful to assess symptom control and quality of life impairment in patients suffering from AR [23]. AR is associated with a vigorous inflammatory response. As a consequence of exposure to an allergen, multiple types of inflammatory cells are recruited to the nasal lining, including B-cells, CD4-positive T-cells, eosinophils, macrophages, and mast cells [24]. Challenged by an allergen, cytokines and mediators are released in the early phase of the immune response, which escalates the cellular inflammatory response over the next four to eight hours. This late phase inflammatory response leads to recurrent symptoms, most commonly nasal congestion.

The production of IgE by plasma cells is stimulated by type 2 T helper cells infiltrating the nasal mucosa, which, when activated, secrete cytokines such as interleukin 3 (IL-3), IL-4, IL-5, and IL-13, which initiate IgE production. IgE goes on to stimulate the secretion of histamine and leukotrienes. These inflammatory mediators cause arteriolar dilation, itching, contraction of smooth muscle, increased vascular permeability, mucous secretion, and rhinorrhea [25].

The findings of our research show that there is a statistically significant relationship between vitamin D deficiency and serum eosinophils. This indicates that there is a significant relationship between vitamin D deficiency and AR. In addition, the results of the study show that there is no statistically significant association between vitamin D deficiency and the patients' gender or age. A study by Arshi et al. generated comparable findings, with the prevalence of severe vitamin D deficiency being significantly greater in AR patients compared to healthy controls [24]. As hypothesized by Wjst and Hyppönen, a possible mechanism for the observed differences between AR patients and healthy controls could lie in aberrant vitamin D metabolism or sensitivity in AR patients [26].

Mostafa et al. found a statistically significant difference in vitamin D levels in groups of allergic fungal rhinosinusitis and chronic rhinosinusitis patients with nasal polyps when compared with patients with chronic rhinosinusitis without nasal polyps and controls (p: <0.001). There were no statistically significant differences between the four groups with regard to calcium levels [27].

The first scientists to hypothesize about the association between the nutritional intake of vitamin D and allergies were Wjst and Dold [28]. They postulated that as vitamin D influences allergy-mediating immune cells in individuals with low 25(OH)D serum levels, the function of T-cells and cellular immune functions that protect against allergies may be affected. Therefore, this hypothesis indicates that vitamin D has a role in the development of allergies. Vitamin D also targets cells that mediate allergy, e.g., eosinophils and mast cells. Mast cell synthesis of IL-10 is increased by cutaneous vitamin D synthesis; IL-10 secretion suppresses skin inflammation.

Since chronic AR is persistent, it presents a treatment challenge to most physicians and otolaryngologists. However, the results of this research offer the promise of improving the outcomes by optimizing vitamin D levels.

## Conclusions

The present study investigated the relationship between vitamin D deficiency and AR, and the effect of vitamin D levels on atopy markers. The data showed that patients with AR had lower serum vitamin D levels. There was a statistically significant relationship between vitamin D deficiency and high levels of eosinophils, but there was no significant relationship between vitamin D deficiency and serum IgE levels. In conclusion, vitamin D deficiency was found to be strongly associated with AR. We strongly recommend further studies with larger sample sizes to provide more insight into the subject.
